# Effects of Hypothermia and Allopurinol on Oxidative Status in a Rat Model of Hypoxic Ischemic Encephalopathy

**DOI:** 10.3390/antiox10101523

**Published:** 2021-09-25

**Authors:** Cristina Durán Fernández-Feijóo, Javier Rodríguez-Fanjul, Miriam Lopez-Abat, Stephanie Hadley, Mónica Cavia-Saiz, Pilar Muñiz, Juan Arnaez, José Ramón Fernández-Lorenzo, Marta Camprubí Camprubí

**Affiliations:** 1 Department of Neonatology, Hospital Álvaro Cunqueiro, EOXI, 36312 Vigo, Spain; cris_dff@hotmail.com (C.D.F.-F.); joseramon.fernandez.lorenzo@usc.es (J.R.F.-L.); 2 Neonatal Intensive Care Unit, Paediatrics Department, Hospital Germans Trias i Pujol, Universitat Autonoma de Barcelona, 08916 Badalona, Spain; javier.rodriguez.fanjul@gmail.com; 3 Department of Neonatology, BCNatal|Barcelona Center for Maternal Fetal and Neonatal Medicine Hospital Sant Joan de Déu and Hospital Clínic, University of Barcelona, 08950 Esplugues de Llobregat, Spain; mlopeza@fsjd.org; 4 Vanderbilt University School of Medicine, Nashville, TN 37232, USA; stephanie.hadley@childrens.harvar.edu; 5 Department of Biotechnology and Food Science, Facutlty of Sciences, University of Burgos, 09001 Burgos, Spain; monicacs@ubu.es (M.C.-S.); pminuiz@ubu.es (P.M.); 6 Department of Neonatology, Hospital Universitario de Burgos, NeNe Foundation, 09006 Burgos, Spain; jusoru@hotmail.com

**Keywords:** allopurinol, hypothermia, hypoxic-ischemic encephalopathy, oxidative stress, oxidative damage

## Abstract

Hypoxic ischemic encephalopathy (HIE) is one of the main causes of morbidity and mortality during the neonatal period, despite treatment with hypothermia. There is evidence that oxidative damage plays an important role in the pathophysiology of hypoxic-ischemic (HI) brain injury. Our aim was to investigate whether postnatal allopurinol administration in combination with hypothermia would reduce oxidative stress (OS) biomarkers in an animal model of HIE. Postnatal 10-day rat pups underwent unilateral HI of moderate severity. Pups were randomized into: Sham operated, hypoxic-ischemic (HI), HI + allopurinol (HIA), HI + hypothermia (HIH), and HI + hypothermia + allopurinol (HIHA). Biomarkers of OS and antioxidants were evaluated: GSH/GSSG ratio and carbonyl groups were tested in plasma. Total antioxidant capacity (TAC) was analyzed in plasma and cerebrospinal fluid, and 8-iso-prostaglandin F2α was measured in brain tissue. Plasma 2,2′–azinobis-(3-ethyl-benzothiazoline-6-sulfonic acid) (ABTS) levels were preserved in those groups that received allopurinol and dual therapy. In cerebrospinal fluid, only the HIA group presented normal ferric reducing ability of plasma (FRAP) levels. Protein oxidation and lipid peroxidation were significantly reduced in all groups treated with hypothermia and allopurinol, thus enhancing neuroprotection in HIE.

## 1. Introduction

Hypoxic ischemic encephalopathy (HIE) is one of the main causes of mortality and long-term disability during the neonatal period [1,2]. Therapeutic hypothermia (TH) is now well established as standard treatment for infants with moderate-to-severe HIE. However, up to 25% of these infants die, and 20 to 60% survive with neurocognitive sequel [3].

Animal models allow for a better understanding of the pathophysiological mechanisms of hypoxic-ischemic brain injury (HI) and play a key role in investigating new therapeutic strategies [4].

HI triggers an increase in free radical production and subsequently oxidative stress (OS). Increased reactive oxygen and nitrogen species alter the function and/or structure of proteins, nucleic acids, and membrane lipids. The newborn brain is particularly susceptible to HI and OS damage due to its immaturity as well as its low concentrations of antioxidant defenses, increased production of superoxide (O_2_^•−^), and a high content of free iron and polyunsaturated fatty acids [5].

There is evidence that oxidative damage plays an important role in the pathophysiology of HI brain injury [6,7]. Martini et al. classified the mechanism of injury in neonatal encephalopathy in three phases [8]: oxygen deprivation in Phase I (0–6 h), mitochondrial dysfunction and increased oxidative stress (lipid, protein, nucleic acid peroxidation) in Phase II (6–72), and epigenetic changes induced by free radicals, inflammation, and decreased neurogenesis in Phase III (>72 h).

Several neuroprotective therapies have been employed to neutralize the excess of free radicals, including therapeutic hypothermia (TH), allopurinol, melatonin, erythropoietin (EPO), and nitric oxide synthase (NOS) inhibitors [7,8,9]. Studies of TH suggest that it may decrease free radical production [10,11]. Similarly, allopurinol, a xanthine oxidase inhibitor and a free radical scavenger, has shown neuroprotective effects against hypoxia-reperfusion brain injury [12,13,14]. To our knowledge, there are no animal studies investigating the combined use of hypothermia and allopurinol as a neuroprotective strategy.

The aim of this study was to investigate whether postnatal allopurinol administration in combination with hypothermia would reduce oxidative stress in an animal model of HIE.

## 2. Materials and Methods

This study was performed following the Guide for the Care and Use of Laboratory Animals of the National Institutes of Health. The protocol was approved by the Ethics Committee for Animal Experimentation of the University of Barcelona (permit number 6575), following European (2010/63(UE)) and Spanish (RD 53/2013) regulations for the care and use of laboratory animals.

All surgeries were performed under inhaled isoflurane (2%), and all efforts were made to minimize the animals’ suffering and the quantity of animals used. Animals were euthanized prior to the end of the experiments by administration of intra-peritoneal thiopental.

### 2.1. Experimental Design

On postnatal day 10 (P10), Wistar pup rats (HARLAM, Netherlands) weighing 12–14 g were used in this study. After birth, animals were kept with their mothers in cages with 12 h light/dark cycles at a constant temperature of 22 ± 1 °C with free access to food and water.

Hypoxia-ischemia (HI) was induced using the Rice–Vannucci model [15]. Seventy-one P10 rat pups underwent left common carotid artery ligation, following the described protocol. After <1 h of recovery with their mother, animals were exposed to 90 min of hypoxia (8% atmospheric oxygen) at 36–36.5 °C rectal temperature, which resulted in HI moderate insult [16,17]. Seven animals died (9.5%) after the procedure prior to being randomized. A total of 64 P10 rat pups were randomized into five experimental groups: sham-operated control (C), HI + normothermia (HI), HI + allopurinol (HIA), HI + hypothermia (HIH), and HI + hypothermia + allopurinol (HIHA).

Sham-operated animals were anesthetized, and a skin incision was performed to expose the left common carotid artery without artery ligation or hypoxia.

#### 2.1.1. Allopurinol

All treatment groups received a single intraperitoneal injection of either allopurinol (zylosprim sodium, Burroughs Wellcome, Research Triangle Park, NC, USA) at 135 mg/kg (volume: 0.01 mL/g; HIA and HIHA groups) or saline (HI and HIH groups) depending on randomization, 15 min after the hypoxia procedure (before beginning the hypothermia or normothermia protocol), as described by Rodriguez-Fanjul et al. [18].

#### 2.1.2. Hypothermia vs. Normothermia

At the end of the HI procedure, pups were also randomized into two groups: (i) those treated with systemic hypothermia (32.5–33 °C) and (ii) those maintained in normothermia (36–36.5 °C). All the pups were maintained in temperature-controlled chambers for 5 h and separated from each other to avoid rewarming. Temperature was continuously measured in one pup in each chamber with a rectal temperature probe (IT-21; Physitemp Instruments). After the treatment period, pups were immediately removed from the chamber and returned to their litter.

**Figure 1:**
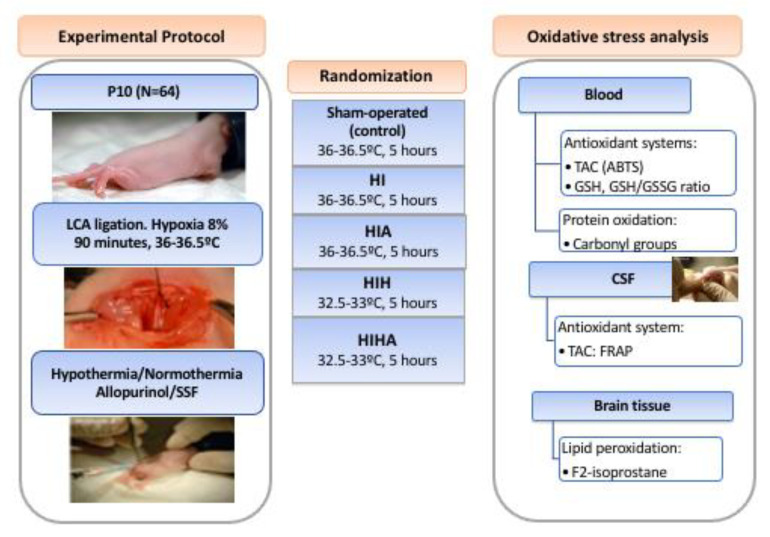
Study Diagram: Experimental Protocol, Randomization, and Oxidative stress analysis. P10: Ten days of life, LCA ligation: Left common carotid ligation. HI: Hypoxia-ischemia, HIA: Hypoxia-ischemia allopurinol, HIH: Hypoxia-ischemia hypothermia, HIHA: Hypoxia-ischemia hypothermia allopurinol. SSF: Physiologic serum.

A diagram of the experimental design, including the number of animals used, is presented in Figure 1.

### 2.2. Samples Obtention and Preparation

Seventy-two hours after the procedure, blood and cerebrospinal fluid (CSF) samples were obtained, processed, and stored at −80 °C until analysis. Pups were sacrificed immediately after sample collection according to protocol, and the brain was removed and stored at −80 °C.

Blood samples: Blood samples were collected in heparin tubes. Plasma was separated by centrifugation (3000× *g* rpm × 10 min) and stored at −80 °C. Hemolyzed blood plus distilled water was treated with cold chloroform: ethanol (3:5 *v*/*v*) and centrifuged (3000× *g* rpm × 10 min), and the supernatant was used for analysis.

CSF samples: CSF collection was performed as previously described [19] and stored at −80 °C.

Brain tissue samples: Coronal sections of 3 mm were cut under dry ice, and the hippocampus and cortico-subcortical area were located and dissected under microscopic visualization (Nikon SMZ645 (16–100 x)). Hippocampus and cortico-subcortical zones were extracted from the brain tissue, homogenized (10% *w*/*w*) in phosphate-buffered saline (PBS) with a pH of 7.4, and centrifuged (2500× *g* rpm × 10 min). The supernatant was then isolated and stored until analysis.

### 2.3. Sample Analysis

#### 2.3.1. Plasma Total Antioxidant Capacity (TAC)

Total antioxidant capacity evaluates the overall antioxidant status of plasma and CSF as an estimation of the plasma capacity to neutralize oxidants. This technique is an overall measure of cumulative antioxidant status of biological fluids instead of individual antioxidants.

The total antioxidant capacity was evaluated in plasma and CSF. The ferric reducing ability power (FRAP) method was used in CSF. Due to the size of the pups, the volume of plasma was small, and some hemolysis was observed. As a result, the 2,2′-azino-bis-3-etilbenzothiazol-6-sulfonic acid (ABTS) method, unaffected by hemolysis, was used to evaluate TAC in plasma.

FRAP assay: This method was used to measure the presence of reducing agents in CSF [20]. This assay measures wavelength absorbance at 593 nm caused by the formation of blue-colored tripyridyl-s-triazine complexes (TPTZ) with ferric (II) [TPTZ-Fe (II)] in the presence of a reducing agent. The results are expressed as molar (mM) equivalents of Fe (II) (mM Fe (II)E). Fe (II) sulfate heptahydrate (FeSO_4_ 7H_2_O) was purchased from Probus S.A. (Badalona, Spain).

ABTS method: This method was used to evaluate the ability of plasma antioxidants to scavenge the ABTS^+^ radical, which absorbs wavelengths at 734 nm. 2,2′-Azino-bis(3-ethylbenzothiazoline-6-sulfonic acid) diammonium salt >98 (ABTS) was performed following the protocol outlined by Re et al. [21] and modified by Gonzalez et al. [22]. Briefly, the ABTS·+ radical cation is generated by the reaction of a 7 mM solution of ABTS in water with 2.45 mM potassium persulphate (K_2_S_2_O_8_) >99% (1:1). The assay consists of 960 μL of ABTS^+^, 35 μL of the buffer PBS 7 mM with a pH of 7.4, and 5 μL of the plasma sample. The inhibition of the absorbance of the ABTS^+^ cation at 734 nm by the sample is measured after a 5 min incubation period. The results are expressed as molar equivalents (mM) of 6-hydroxy-2,5,7,8-tetramethylchroman-2-carboxylic acid 97% (TROLOX).

#### 2.3.2. Plasma Glutathione Reduced/Oxidized (GSH/GSSG) Ratio Analysis

Plasma reduced glutathione (GSH) and oxidized glutathione (GSSG) levels were determined using the reaction between the sulfhydryl group of the GSH and 5,5′-dithio-bis-2-nitrobenzoic acid (DTNB, Ellman’s reagent) [23]. The GSTNB (mixture between GSH and TNB) is reduced by glutathione reductase to recycle GSH and produce more TNB. The levels of TNB produced were directly proportional to the concentration of GSH in the sample. Briefly, the extract of hemolyzed blood was mixed with DTNB, NADPH, and glutathione reductase (Sigma-Aldrich, St. Louis, MO, USA). The level of total GSH (reduced and oxidized) was evaluated by measuring absorbance at 412 nm at 2 min intervals for 20 min. Glutathione disulfide (GSSG) was measured using the same method after derivatizing the samples with 2-vinilpiridine, and GSH was estimated by subtracting GSSG from total GSH. The results are expressed as the GSH/GSSG ratio.

#### 2.3.3. Plasma Protein Carbonyl Groups (CG)

Plasma protein oxidation was assessed with an estimation of carbonil groups formed using the protocol described by Levine et al. [24] which is based on the reaction of the carbonyl group with 2,4-dinitrophenylhydrazine (DNFH) under acidic conditions. Plasma samples were mixed with DNFH and incubated 1 h. After that, they were precipitated with 500 μL of 20% (*w/v*) of trichloroacetic acid, washed three times with ethanol/ethyl acetate (1:1 *v/v*), and centrifuged at 6000× *g* for 3 min to remove any free 2,4-DNPH. Finally, 1 mL of 6 M guanidine, pH 2.3, was added at the samples and were incubated in a 37 °C water bath for 30 min. Carbonyl groups were calculated by absorption spectrophotometry at 373 nm, using a molar absorption coefficient of 22,000 M^−1^ cm^−1^. The carbonyl groups levels were normalized by the protein concentration in plasma, and the results are expressed as nmol/mg protein. Total protein concentration was determined by the Lowry method.

#### 2.3.4. Brain 8-Iso-Prostaglandin F2α (8-iso-PGF2α) Quantification

Levels of 8-iso-PGF2α were measured in the hippocampus and cortico-subcortical area 72 h after the HI event. They were quantified by ELISA according to kit instructions (OxiSelect 8-iso-Prostaglandin F2α ELISA Kit). To be prepared for the analysis, samples were treated with NaOH at 45 °C for 2 h. In addition, 100 μL of concentrated (10N) HCl per 500 μL of hydrolyzed sample was added. After that, samples were centrifugated for 5 min at 12,000× *g* rpm.

### 2.4. Statistical Analysis

Results are expressed in mean and interquartile range. The Kruskal–Wallis test was used to detect differences between groups. Post hoc analysis was performed to evaluate inter-group differences. STATA v13 was utilized for statistical analysis.

## 3. Results

A total of 71 rat pups were used for this experiment. Seven (9.5%) died after the procedure. None of the animals treated with HIH or HIHA died. All the samples were obtained 72 h after the procedure. The mean animal weight was 19.2 ± 3.7 g. From each animal a maximum of 0.5–0.7 mL of total blood was obtained, and this was immediately processed to one of the experiments, with the goal of having enough representative samples of each biomarker. Due to the small blood sample, not all of the animals were tested for all the biomarkers.

### 3.1. Allopurinol Administration, Alone or in Combination with Hypothermia, Protects Total Antioxidant Capacity (TAC) after an HI Event

**Figure 2:**
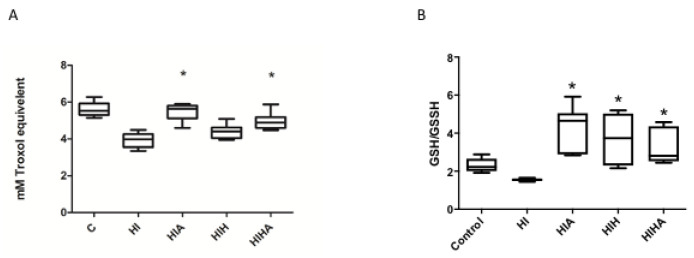
Antioxidant systems: (**A**) Box plot plasma ABTS levels; (**B**) Box plot plasma GSH/GSSG ratio. C: Control, HI: Hypoxia-ischemia, HIA: Hypoxia-ischemia + allopurinol, HIH: Hypoxia-ischemia + hypothermia, HIHA: Hypoxia-ischemia + hypothermia + allopurinol. * *p* < 0.05 in post-hoc analysis compared to HI.

Plasma TAC levels measured by ABTS were lower in the HI group compared to HIA and HIHA (*p* < 0.001), but there were no differences found between HI and those animals treated only with hypothermia (HIH) (*p* = 0.194). Mean plasma TAC values are presented in Table S1 (Supplementary Materials). In the post hoc analysis, differences were also seen between HIHA and HIH groups (*p* < 0.001) (Figure 2A).

### 3.2. GSH/GSSG Ratio Was Decreased after an HI Event, and Only Treated Groups Recovered to Normal Values

The plasma GSH/GSSG ratio was decreased in HI animals compared to those groups that received any treatment (HIA, HIH, HIHA) (*p* < 0.001). Levels in the HIA group were even higher than those in the C group (*p* < 0.001), as shown in Table S2 (Supplementary Materials) (Figure 2B).

### 3.3. TAC Levels in CSF Are Preserved When Allopurinol Is Administrated after an HI Event, but Not When Allopurinol Is Administrated in Combination with Hypothermia

**Figure 3:**
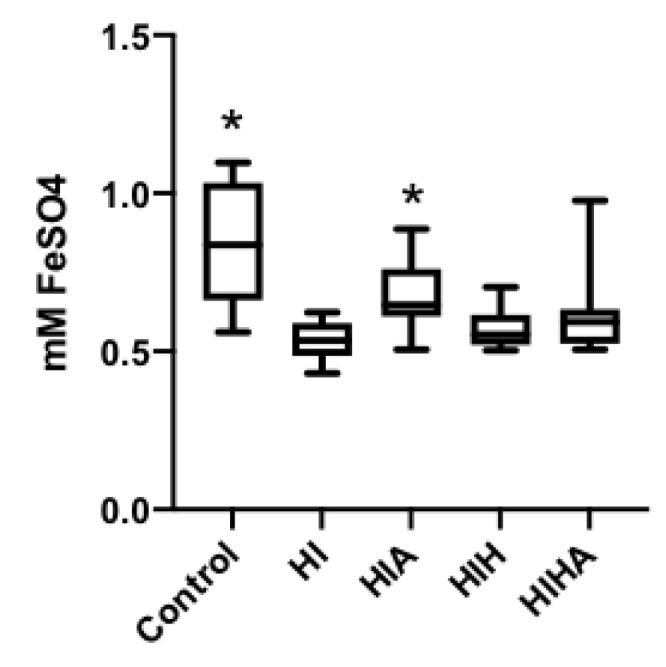
Box plot cerebral spinal fluid FRAP levels. C: Control, HI: Hypoxia-ischemia, HIA: Hypoxia-ischemia + allopurinol, HIH: Hypoxia-ischemia + hypothermia, HIHA: Hypoxia-ischemia + hypothermia + allopurinol. * *p* < 0.05 in post-hoc analysis compared to HI.

HI animals showed the lowest levels of TAC in the CSF, as quantified by the FRAP method (*p* = 0.011). The HIA group presented similar levels to the C group (*p* = 0.834). No increases in TAC levels were detected in hypothermia-treated groups (HIH, HIHA). Results are presented in Table S3 (Supplementary Materials) (Figure 3).

### 3.4. Protein Oxidation Increase after an HI Event, All Treatments Seem to Prevent Protein Oxidation

**Figure 4:**
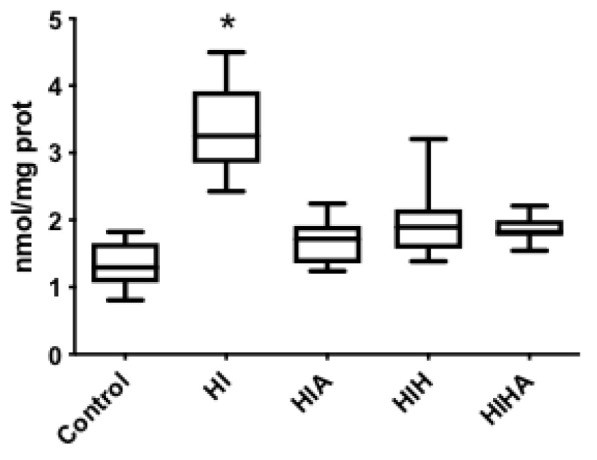
Box plot plasma carbonyl groups. C: Control, HI: Hypoxia-ischemia, HIA: Hypoxia-ischemia + allopurinol, HIH: Hypoxia-ischemia + hypothermia, HIHA: Hypoxia-ischemia + hypothermia + allopurinol. * *p* < 0.05 in post-hoc analysis compared to HI.

Plasma carbonyl group levels were significantly increased in the HI group. In the post hoc analysis, treated groups (HIA, HIH, HIHA) presented with lower plasma carbonyl group levels compared to HI (*p* < 0.01). Results are presented in Table S4 (Supplementary Materials) (Figure 4).

### 3.5. Lipid Peroxidation Increased after an HI Event and Decreased in the Treated Groups

**Figure 5:**
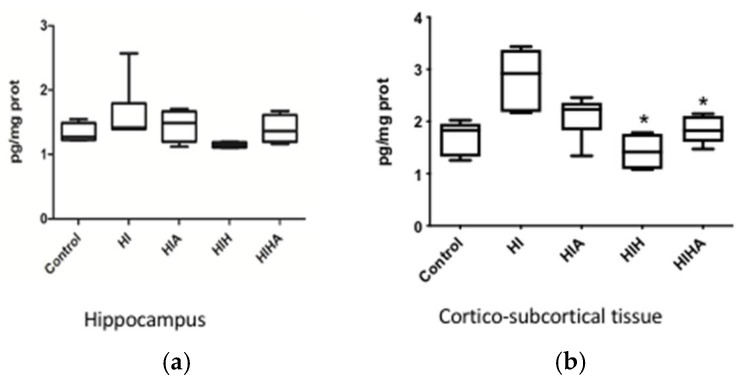
(**a**) Box plot of F2-isoprostane levels in hippocampus tissue. (**b**) Box plot of F2-isoprostane levels in cortico-subcortical tissue. C: Control, HI: Hypoxia-ischemia, HIA: Hypoxia-ischemia + allopurinol, HIH: Hypoxia-ischemia + hypothermia, HIHA: Hypoxia-ischemia + hypothermia + allopurinol. * *p* < 0.05 in post-hoc analysis compared to HI.

Hippocampal 8-iso-PGF2α levels varied among groups (*p* = 0.030) (Figure 5a). In the cortical-subcortical area, 8-iso-PGF2α levels increased in the HI group (*p* = 0.002) and significantly decreased in HIH (*p* < 0.001) and HIHA (*p* = 0.013) (Figure 5b). Table S5 (Supplementary Materials).

## 4. Discussion

There is increasing evidence supporting the role of oxidative stress in the pathogenesis of brain damage in HIE. Many studies have examined antioxidant therapies for treatment and have demonstrated their neuroprotective effect [18,25,26,27] despite their mechanism of action being unclear. In our previous paper, hypothermia and allopurinol were proven to provide a neuroprotective effect, improving histological, biochemical, and functional markers in a neonatal animal model of HIE [18]. In the same line, there is one ongoing clinical trial that is also studying the effect of allopurinol in addition to hypothermia in neonates with HI brain injury on neurocognitive outcome [28]. Despite these studies, nothing has been published about its effects on antioxidant systems and free radical production when hypothermia and allopurinol are administered together as a combined neuroprotective strategy.

After an HI event, a decrease in serum TAC levels was observed. These results are in agreement with the findings of other publications that demonstrate a reduction in TAC levels in cerebral tissue following HI injury [29,30]. In our study, TAC levels were preserved after the administration of the neuroprotective therapy with allopurinol, or the combination therapy of hypothermia and allopurinol. Curiously, Van Bel and colleagues, in their clinical trial using allopurinol in HIE patients, could not demonstrate this protective effect of allopurinol in relation to TAC levels. They hypothesized that this could be the result of the delayed administration of allopurinol (170 min after the insult), highlighting the importance of early administration of the antioxidant therapies after an HI insult [14]. Experimental studies have demonstrated that while free radical production begins with ischemia, it significantly increases during the reperfusion phase [31]. Ono and colleagues demonstrated that early administration of allopurinol decreased superoxide radical generation [32]. Another important point that supports early administration of allopurinol is related to the results observed in CSF TAC levels. Allopurinol is able to cross the blood brain barrier (BBB) [33]. Of note, while HIE increases BBB permeability [34], hypothermia causes its stabilization [35]. Analyzing CSF in our population, only the allopurinol group (HIA) maintained TAC levels similar to the control group, perhaps attributable to this BBB stabilization. These results support the perception that allopurinol must be administered as soon as possible before hypothermia, leading to BBB stabilization and, subsequently, difficult passage of allopurinol into the CNS. In this regard, Kaandorp JJ et al. presented the ALLO trial, in which allopurinol was administered antenatally when hypoxia was suspected [36]. In this study, a decrease in a biomarker of brain damage (S100B protein) was reported in the allopurinol group. Recently, based on the importance of the early allopurinol administration after an HI event, there is an ongoing multicenter trial (Phase III) studying the neuroprotection effect of allopurinol in addition to hypothermia treatment (ALBINO trial) in newborns with hypoxic-ischemic encephalopathy. In this study, allopurinol was administered intravenously within 30 min after birth to optimize the timing and inhibition of superoxide formation in asphyxiated infants with evolving HIE [28].

GSH is a critical non-enzymatic antioxidant protecting cells from OS. In our population, the GSH/GSSG ratio was decreased in the HI group. These data are in accordance with other published results [35], supporting the hypothesis that after an HI event there is a massive liberation of free radicals that consume reduced glutathione. Of note, groups undergoing neuroprotective treatments showed similar GSH/GSSG ratios as the control group. Moreover, it has been demonstrated that allopurinol plays a role in GSH normalization levels in experimental models of ischemia/reperfusion, renal failure, and respiratory distress [37,38,39]. In our study, GSH/GSSG ratios in the HIA group were remarkably higher, even higher than those of controls. This result opens a path to explore whether or not allopurinol, similar to other antioxidants such as melatonin [25], may have a role in the induction of antioxidant substances. Regarding hypothermia treatment, we observed a protective effect on the GSH/GSSG ratio consistent with previously described experimental models of cardiac circulatory arrest or cerebral hypoxia where hypothermia was induced [40,41]. In the same line, neuroprotection in terms of histology, biomarkers, and function were also demonstrated in our previous paper [18], where all the treatment groups presented an improvement in all of these aspects after treatment administration.

Protein oxidation and lipid peroxidation were also evaluated in our HIE model. Protein oxidation in HIE, measured by serum carbonyl group levels, has been previously proven in clinical and experimental essays [42,43,44], supporting our findings in the HI group. In our study, administration of any neuroprotective treatment (HIA, HIH, HIHA) seemed to prevent protein oxidation. Experimental studies have demonstrated that hypothermia decreases the degree of protein oxidation 3 to 6 h after an HI event [42,45]. It is not well known how allopurinol protects against protein oxidation, but this effect has also been described in heart disease studies [46].

Increased lipid peroxidation has also been reported after an HI event. There are many biomarkers used to evaluate this status. For example, 8-iso-PGF2α, a widely used OS biomarker [47], was analyzed in the hippocampus and cortico-subcortical zones. The elevated levels of 8-iso-PGF2α in the HI group are in accordance with other publications, demonstrating increases in lipid peroxidation products in the cortex and hippocampus in a neonatal rat model of HIE [48]. Lower levels of 8-iso-PGF2α were observed in all treated groups. Allopurinol effects on lipid peroxidation are not well established and, to our knowledge, have not been studied in HI animal models. However, it appeared to have a protective role in clinical cardiovascular trials, similar to its protection against protein oxidation [49].

There is also controversy regarding the effects of hypothermia on lipid peroxidation. Recently, Huun and colleagues demonstrated in a porcine animal model of HIE a decrease in urinary levels of 8-iso-PGF2α in those animals that were treated with hypothermia, but none of the other lipid peroxidation compounds evaluated were affected by the hypothermia treatment [50]. This group also analyzed lipid peroxidation in the cortex, subcortical white matter, and hippocampal tissue, finding a decrease in lipid peroxidation products only in subcortical white matter [51].

In our previous paper, Rodríguez-Fanjul J et al. [18], hypothermia, allopurinol + hypothermia, and allopurinol therapies were proven to confer neuroprotection after an HI event. Functional, histologic, and molecular improvement were described in all treated groups using the same protocol study. Histologically, damaged area and hippocampal volume were different among treatment groups. The largest tissue lesions were observed in the HI group, followed by HIA. From a molecular point of view, cleaved caspase 3 expression was increased in both HI and HIA. These results are in accordance with the decrease in the oxidative stress biomarkers that we observed in the HIHA and HIH groups. In the present study we also detected some positive effect against oxidative stress in the HIA group, reflecting the antioxidant effect of allopurinol. In fact, from a functional point of view, as shown in our previous paper [18], animals undergoing neuroprotection therapies, including the HIA group, presented an improvement in short-term (negative geotaxis) and long-term (Water Morris test) functional tests. Moreover, when the learning process was analyzed, no differences were found between treated groups. Animals from the HIA group had similar results to the HIH and HIHA groups.

Our paper reinforces the notion that the newborn brain is vulnerable to oxidative stress after an HI event. When considering all our data, it can be concluded that the administration of allopurinol, hypothermia, and the combination treatment (hypothermia + allopurinol) protects the brain against oxidative damage.

We observed changes in the expression of several oxidative stress biomarkers from different sources (serum, cerebral tissue, and CSF), and all of them support our previously histological biochemical and functional published results [18].

Finally, our data may also reinforce the importance of early administration of allopurinol treatment, even before initiating hypothermia, to achieve an optimal concentration in the CSF and improve and avoid an increased oxidative stress response. We believe that this finding may be imperative for future clinical trials of HIE that utilize allopurinol as a neuroprotective agent.

## Data Availability

Data is contained within the article and Supplementary Materials.

## Supplementary Materials

The following are available online at https://www.mdpi.com/article/10.3390/antiox10101523/s1. Table S1. Plasma TAC levels measured using ABTS, Table S2. Plasma GSH/GSSG ratio, Table S3. Cerebral Spinal Fluid TAC levels, Table S4. Plasma carbonyl group levels, Table S5. Lipid peroxidation.
