# Supplementary material for: Effects of Hypothermia and Allopurinol on Oxidative Status in a Rat Model of Hypoxic Ischemic Encephalopathy
Source: Antioxidants (Basel). 2021 Sep 25;10(10):1523. doi: 10.3390/antiox10101523 (PMC8533154; doi:10.3390/antiox10101523)
Supplement: Supplementary file 1 [file antioxidants-10-01523-s001.zip › antioxidants-1393201-supplementary.pdf]

Table S1. Plasma TAC levels measured using ABTS.

ABTS (TEACmM)	
**Group (n=62)**	Mean [IQ95%]
C (n=12)	5.549 [5.32–5.92]
HI (n=10)	3.959 [3.42–4.26]
HIA (n=10)	5.63 [5.11–5.81]
HIH (n=13)	4.41 [4.04–4.62]
HIHA (n=17)	4.88 [4.62–5.05]

Mean [Interquartile range] C: Control, HI: Hypoxia–ischemia, HIA: Hypoxia–ischemia + allopurinol, HIH: Hypoxia–ischemia + hypothermia, HIHA: Hypoxia–ischemia + hypothermia + allopurinol.

Table S2. Plasma GSH/GSSG ratio.

Ratio **GSH/GSSG**	
**Groups (n=36)**	**Median [IQ95%]**
C (n=9)	2.23 [1.99–2.67]
HI (n=6)	1.55 [1.45–1.64]
HIA (n=7)	4.65 [2.85–5.65]
HIH (n=7)	3.73 [2.20–5.14]
HIHA (n=7)	2.80 [2.47–4.50]

Mean [Interquartile range] C: Control, HI: Hypoxia–ischemia, HIA: Hypoxia–ischemia + allopurinol, HIH: Hypoxia–ischemia + hypothermia, HIHA: Hypoxia–ischemia + hypothermia + allopurinol.

Table S3. Cerebral Spinal Fluid TAC levels.

**FRAP CSF (mMFeSO4)**	
**Groups (n=41)**	**Median [IQ95%]**
C (n=9)	0.837 [0.647–1.07]
HI (n=9)	0.533 [0.485–0.601]
HIA (n=9)	0.646 [0.588–0.792]
HIH (n=6)	0.552 [0.505–0.691]
HIHA (n=8)	0.592 [0.51–0.75]

Mean [Interquartile range] C: Control, HI: Hypoxia–ischemia, HIA: Hypoxia–ischemia + allopurinol, HIH: Hypoxia–ischemia + hypothermia, HIHA: Hypoxia–ischemia + hypothermia + allopurinol.

Table S4. Plasma carbonyl group levels.

Carbonyl groups (nmol/gprot)	
**Groups (n=64)**	**Median [IQ95%]**
C (n=12)	1.29 [1.06–1.67]
HI (n=12)	3.25 [2.83–3.94]
HIA (n=12)	1.71 [1.35–1.91]
HIH (n=13)	1.89 [1.559–2.186]
HIHA (n=15)	1.82 [1.77–1.99]

Mean [Interquartile range] C: Control, HI: Hypoxia–ischemia, HIA: Hypoxia–ischemia + allopurinol, HIH: Hypoxia–ischemia + hypothermia, HIHA: Hypoxia–ischemia + hypothermia + allopurinol.

Table S5. Lipid peroxidation.

**8–iso–PGF2α levels (pg/mg protein)**		
**Groups (n=26)**	**Hippocampal Median [IQ95%]**	**Cortical–subcortical area Median [IQ95%]**
C (n=5)	1.27 [1.21–1.54]	1.64 [1.25–2.02]
HI (n=6)	1.41 [1.39–2.46]	2.92 [2.17–3.43]
HIA (n=6)	1.48 [1.13–1.70]	2.23 [1.40–2.44]
HIH (n=4)	1.14 [1.1–1.19]	1.41 [1.07–1.78]
HIHA (n=5)	1.36 [1.16–1.67]	1.82 [1.47–2.14]

Mean [Interquartile range] C: Control, HI: Hypoxia–ischemia, HIA: Hypoxia–ischemia + allopurinol, HIH: Hypoxia–ischemia + hypothermia, HIHA: Hypoxia–ischemia + hypothermia + allopurinol.
